# Robotic-assisted endoscopic submucosal dissection: a scoping review of preclinical and early clinical evidence

**DOI:** 10.1007/s00464-026-12980-6

**Published:** 2026-07-01

**Authors:** Said Alyacoubi, Kunal Rajput, Mark Runciman, Ara Darzi, Christopher J. Peters, George Mylonas

**Affiliations:** 1 Department of Surgery & Cancer, St. Mary’s Hospital, Imperial College London, 10Th Floor, Queen Elizabeth the Queen Mother Building, Praed Street, London, W2 1NY UK; 2 Department of Surgery & Cancer, Imperial College London, London, UK

**Keywords:** Endoscopic submucosal dissection, Robotic endoscopy, Robotic-assisted ESD, Endoscopic robotics, Early gastrointestinal cancer

## Abstract

**Background:**

Endoscopic submucosal dissection (ESD) is technically demanding and associated with a steep learning curve and increased complication risk. Constraints of conventional endoscopes, together with the observed benefits of robotic assistance in selected surgical procedures, have driven development of robotic systems for advanced endoscopic applications. This review maps the landscape of robotic endoscopic systems in the context of ESD.

**Methods:**

A PRISMA-ScR-compliant scoping review was conducted. Six databases were searched in 2025. Studies evaluating robotic endoscopic platforms for ESD in preclinical or clinical settings were included. Two reviewers independently screened studies. Data were extracted on study characteristics, platform features, experimental design, and outcomes. Findings were synthesised descriptively.

**Results:**

Twenty-seven studies were published between 2010 and 2025, with most from 2019 onwards (22; 82%), and mainly from East Asia (23; 85%). Most studies were preclinical (21; 78%), corresponding to mid-level technological readiness (TRL 4–7). Six studies (22%) reported early clinical evaluation (TRL 8), including one multicentre randomised trial. The most frequently studied site was the stomach (18; 67%). Over half of the studies were comparative (15; 56%), including 14 comparing robotic-assisted ESD (R-ESD) with conventional ESD (C-ESD). In preclinical comparisons (*n* = 13), R-ESD was associated with shorter submucosal dissection times and higher dissection speeds. *En bloc* resection rates were high overall, with lower rates observed mainly in novice-performed C-ESD. Perforation was uncommon overall, with higher rates concentrated in novice-performed C-ESD. Early clinical studies (IDEAL 1–3) demonstrated high *en bloc* resection rates with acceptable safety.

**Conclusion:**

Preclinical comparative evidence suggests improved procedural efficiency with robotic assistance, particularly in novice-operated settings. Early clinical evidence indicates feasibility and acceptable safety. However, findings should be interpreted as exploratory signals rather than established clinical superiority. Further technological refinement and robust clinical evaluation are required before wider adoption.

**Graphical abstract:**

**Figure:**
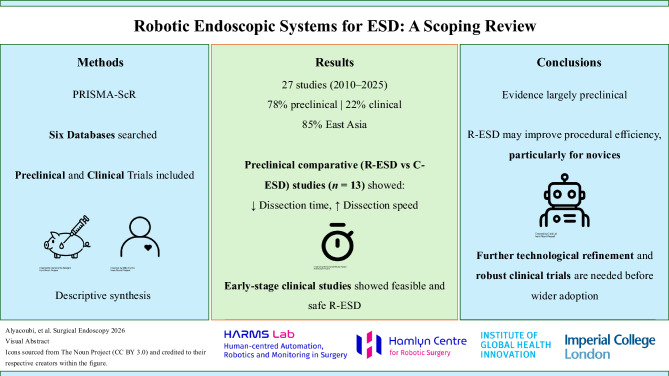

**Supplementary Information:**

The online version contains supplementary material available at 10.1007/s00464-026-12980-6.

Endoscopic submucosal dissection (ESD) enables *en bloc* resection of precancerous lesions and early-stage cancers of the gastrointestinal (GI) tract [1]. Compared with endoscopic mucosal resection (EMR), ESD offers superior curative resection rates, improved histopathological assessment, and lower recurrence [2]. Despite these advantages, ESD is technically demanding, with longer procedure times and higher complication risk. Although widely adopted in Asia, its uptake in Western practice has been slower [3].

ESD requires deliberate, precise, layer-specific dissection and follows the same core principles of conventional surgery [4, 5]. However, the inherent limitations of standard endoscopes prevent the application of effective countertraction, making optimal submucosal exposure difficult to maintain [6]. Several variants of conventional ESD (C-ESD)—such as tunnelling, pocket-creation and hybrid techniques—have been developed to circumvent these limitations [7]. Yet these approaches remain largely passive and cannot replicate the dynamic, adjustable tissue manipulation required for efficient dissection.

Given this unmet need, innovations have focussed on accessory design and traction techniques to emulate the function of a surgeon’s second hand. Meta-analyses consistently demonstrate that traction-assisted ESD leads to shorter procedure times and faster dissection speeds compared to C-ESD [8]. Traction also appears to reduce technical difficulty and support endoscopists during their early stages of training [9, 10]. A wide array of traction methods has consequently emerged, broadly categorised as external (e.g. clip-and-snare, external forceps, magnetic anchors) and internal systems (e.g. S–O Clip, clip-band, multiloop devices). Nevertheless, all existing methods have practical limitations, and none reliably offers stable, directional, and controllable traction across the full spectrum of ESD scenarios [11, 12].

Against this backdrop, increasing attention has turned towards the development of robotic endoscopic platforms capable of providing triangulation, active countertraction, enhanced visualisation, and improved ergonomics [13, 14]. Beyond ESD, such systems have potential applications in other advanced endoscopic procedures, including endoscopic full-thickness resection (EFTR) and natural orifice transluminal endoscopic surgery (NOTES) [15, 16]. Given the technical complexity and steep learning curves associated with these interventions, robotic assistance may reduce procedural demands, accelerate skill acquisition, and facilitate wider adoption across varying levels of endoscopic expertise [17].

## Objectives

The primary objective of this review is to map the landscape of robotic endoscopic systems in the context of ESD. This review additionally seeks to

- Characterise the contexts in which these systems have been evaluated.
- Assess technological maturity using the technology readiness level (TRL) and IDEAL frameworks [18, 19].
- Summarise reported outcomes and explore trends between robotic-assisted ESD (R-ESD) and C-ESD.
- Evaluate the feasibility and safety of R-ESD in early clinical evidence.
- Identify knowledge gaps and future priorities

## Methods

### Review design

This review followed the Arksey–O’Malley framework and the Joanna Briggs Institute (JBI) Manual for Evidence Synthesis (2024). Reporting adhered to the PRISMA-ScR (Preferred Reporting Items for Systematic Reviews and Meta-Analyses extension for Scoping Reviews) checklist. Although initially planned as a systematic review, the predominantly preclinical, heterogeneous and exploratory evidence necessitated a scoping approach.

### Eligibility criteria

Studies were eligible for inclusion if they investigated robotic endoscopic systems applied to ESD in either clinical or preclinical settings. Preclinical studies included porcine-derived ex vivo specimens (excised stomach or colon) or in vivo porcine models (live pigs), provided the experimental setup allowed realistic procedural evaluation.

To ensure procedural relevance, studies using artificial models, non-porcine animal models, or simplified benchtop setups that did not replicate the procedural complexity of ESD were excluded. In addition, studies were required to report at least one of the following outcomes: resected specimen area, submucosal dissection time, submucosal dissection speed, *en bloc* resection rate, or perforation rate.

Non-original publications, abstract-only records, studies evaluating robotic systems for procedures other than ESD, and non-English publications were excluded.

### Information sources and search strategy

A literature search was conducted in Ovid MEDLINE, Embase, Cochrane Library, Web of Science, Scopus, and IEEE Xplore. The initial search was performed between 19 and 20 February 2025. The search was re-run on 28–29 October 2025 using the same search strategy to ensure capture of recent publications. Search strategies were developed with support from an academic librarian. Full search strategies for each database are provided in Supplementary File 1.

### Selection of sources of evidence

Records were deduplicated in Covidence (Veritas Health Innovation, Melbourne, Australia). Title and abstract screening, followed by full-text review, was performed independently by two reviewers (S.A. and K.R.). Discrepancies were resolved by consensus. Reference lists of included studies were hand-searched to identify any additional eligible articles.

### Data charting and data items

Data were charted using a structured Excel spreadsheet aligned with the review aims and piloted on an initial subset of studies. One reviewer (S.A.) extracted data on study characteristics (authors, institutions, country, and publication details), experimental setup (study design, target organ, number of specimens or animals, number of ESD procedures, lesion characteristics, operator experience, comparators, and robotic system evaluated), and procedural outcomes (as predefined in the eligibility criteria), and study-reported key findings and limitations. No formal independent verification of extracted outcome data was performed. However, TRL and IDEAL stage assignments were independently reviewed by a co-author (M.R.) to ensure consistency. Given the descriptive nature of the synthesis and the absence of pooled quantitative analysis, the risk of extraction bias materially influencing the overall conclusions is considered limited. For multi-country studies, country was assigned based on the first author’s primary affiliation.

### Synthesis of results

Evidence was synthesised descriptively. Study characteristics were summarised using SPSS (IBM SPSS Statistics, Armonk, NY, USA). Robotic platforms were grouped by system and summarised according to experimental context and anatomical application. Technological maturity was assessed using the TRL framework for preclinical studies and the IDEAL framework for clinical studies. Technical specifications were synthesised qualitatively across platforms. Comparative preclinical outcomes were synthesised by summarising outcome ranges for R-ESD and C-ESD separately, with direction of effect noted where paired comparisons were available. Pooled analysis was not conducted due to heterogeneity. Clinical studies were summarised individually by reporting key procedural and safety outcomes.

## Results

### Study selection

**Fig. 1:**
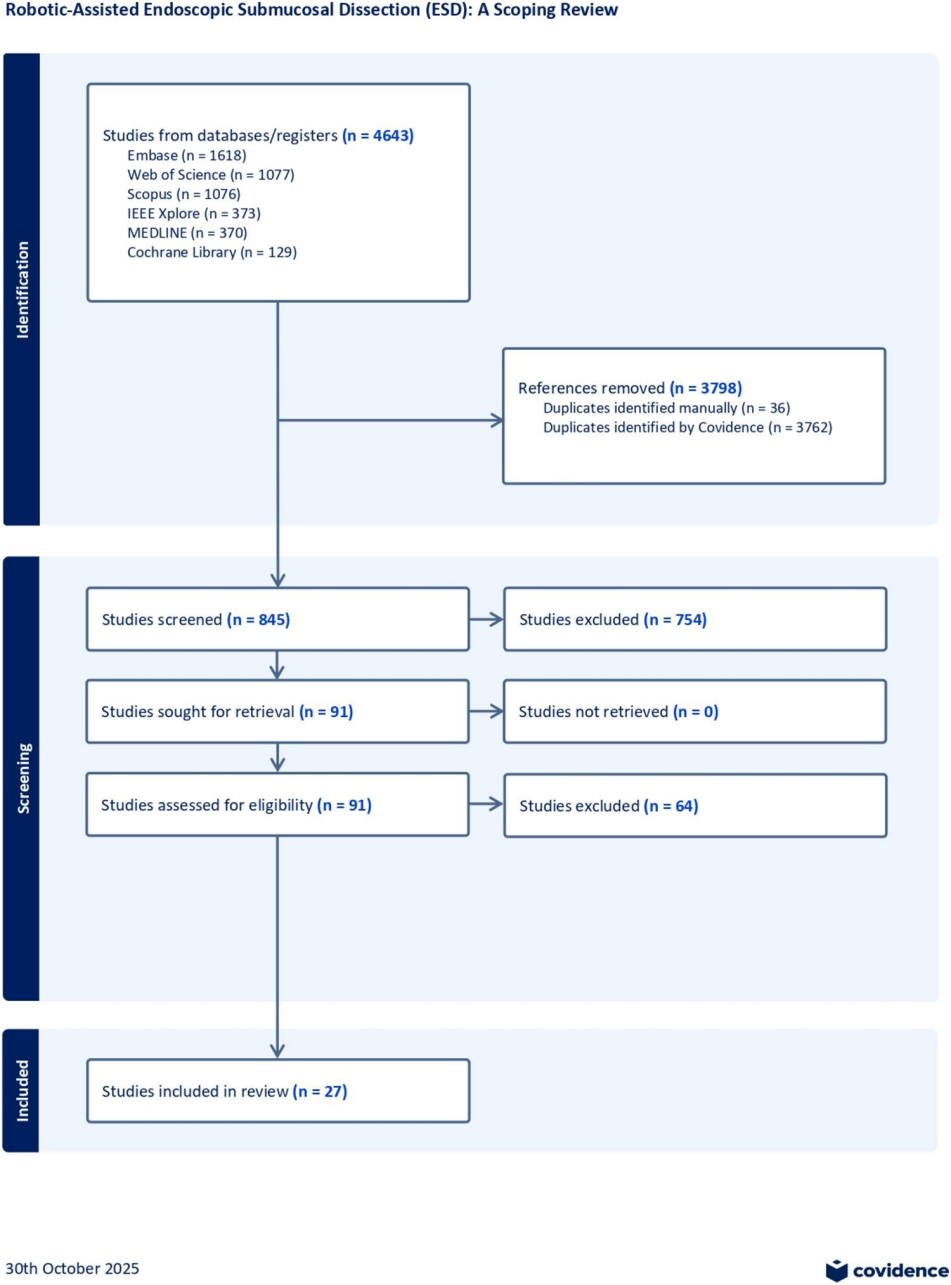
PRISMA-ScR flow diagram showing study identification, screening, eligibility assessment, and inclusion

A total of 4643 records were imported for screening, of which 845 unique records remained after duplicate removal. Following title and abstract screening, 91 studies underwent full-text assessment, and 27 studies met the eligibility criteria and were included. The study selection process is shown in the PRISMA-ScR flow diagram (Fig. 1).

### Study characteristics

**Fig. 2:**
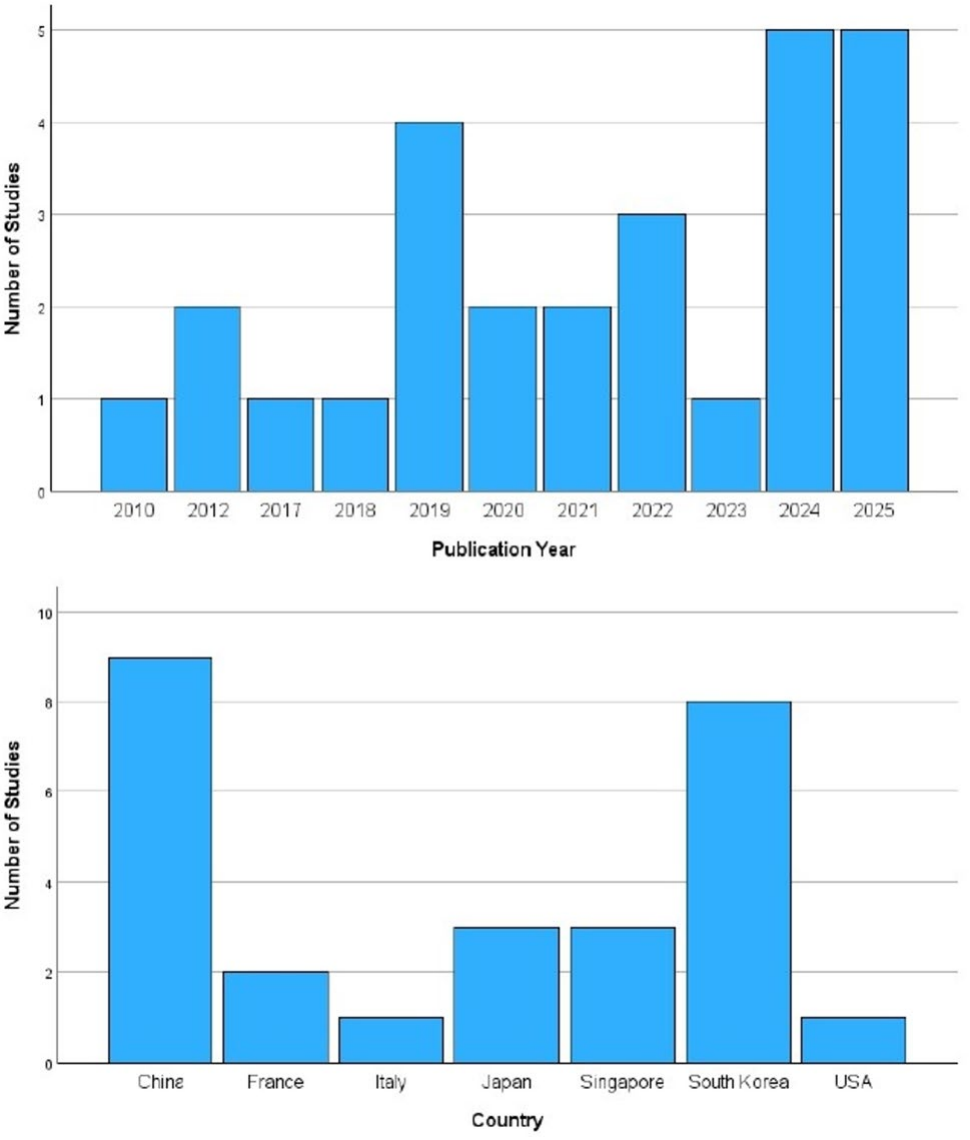
Distribution of included studies by year of publication (top) and country of origin based on first author affiliation (bottom)

A summary of the 27 included studies is presented in Table 1 [20–46]. Studies were published between 2010 and 2025, with a marked increase in output from 2019 onwards, peaking in 2024–2025 (Fig. 2). Most studies originated from East Asia, primarily China (9, 33.3%) and South Korea (8, 29.6%), with fewer contributions from Japan, Singapore, and Western centres (Fig. 2). Overall, 21 studies (77.8%) were preclinical, comprising 8 ex vivo (29.6%), 11 in vivo (40.7%), and 2 combined (ex vivo*/*in vivo*)* experiments (7.4%). The remaining 6 studies (22.2%) were clinical and predominantly representing early-phase feasibility work. Only one multicentre randomised controlled trial (RCT) was identified.

**Table 1:** Characteristics of included studies evaluating robotic endoscopic platforms for ESD

Authorship	Year	Origin of study	Study design	Robotic platform	Anatomical location	Comparative*
Ho et al.	2010	Singapore	ex vivo* & *in vivo	MASTER	Stomach	Yes^†^
Wang et al.	2012	Singapore	in vivo	MASTER	Stomach	No
Phee et al.	2012	Singapore	clinical	MASTER	Stomach	No
Légner et al.	2017	France	in vivo	STRAS v2	Colon/Rectum	No
Iwasa et al.	2018	Japan	ex vivo	RAFE	Stomach	Yes
de Moura et al.	2019	USA	ex vivo	FLEX	Colon	Yes
Kim et al.	2019	South Korea	ex vivo	REXTER	Stomach	Yes
Kume et al.	2019	Japan	ex vivo	ETRS	Stomach	No
Mascagni et al.	2019	France	in vivo	EASE	Colon	Yes
Hwang et al.	2020	South Korea	ex vivo	PETH	Stomach	Yes
Nakadate et al.	2020	Japan	ex vivo	RAFE	Stomach	No
Chiu et al.	2021	China	in vivo	EndoMaster EASE	Colon/Rectum	No
Kim et al.	2021	South Korea	in vivo	ROSE	Stomach	Yes^‡^
Yang et al.	2022	China	ex vivo	FASTER	Stomach	Yes
Ji et al.	2022	China	in vivo	FASTER	Oesophagus & Stomach	Yes
Morino et al.	2022	Italy	Clinical	FLEX	Rectum	No
Yang et al.	2023	China	ex vivo	FASTER	Stomach	Yes
Kim et al.	2024	South Korea	in vivo	PETH	Stomach	Yes
Yang et al.	2024	China	in vivo	DREAMS	Oesophagus & Stomach	Yes
Kim et al.	2024	South Korea	ex vivo* & *in vivo	Non-specified	Colon/Rectum	Yes^§^
Liang et al.	2024	China	in vivo	GIFTS	Oesophagus & Colon/Rectum	No
Gao et al.	2024	China	in vivo	DREAMS	Stomach	No
Kim et al.	2025	South Korea	in vivo	ROBOPERA	Stomach	Yes
Chiu et al.	2025	China	Clinical	EndoMaster EASE	Colon/Rectum	No
Kim et al.	2025	South Korea	Clinical	ROBOPERA	Colon/Rectum	No
Fan et al.	2025	China	Clinical	EndoFaster	Oesophagus & Stomach	Yes
Jeon et al.	2025	South Korea	Clinical	ROBOPERA	Stomach	No

^*^Comparative study defined as robotic-assisted ESD vs conventional ESD, unless otherwise specified

^†^Comparative only in one component of a mixed ex vivo*/*in vivo study (in vivo part)

^‡^Comparison between expert and novice operators using the same robotic endoscopic platform

^§^Comparative only in one component of a mixed ex vivo/in vivo study (ex vivo part)

Fifteen studies (55.6%) were comparative and 12 (44.4%) were non-comparative. Amongst comparative studies, 14 compared R-ESD with C-ESD, and one compared expert versus novice performance using the same robotic platform. Of the 14 R-ESD versus C-ESD comparative studies, one was clinical and 13 were preclinical. Overall, the stomach was the most frequently investigated site (18 studies, 66.7%), including three that also involved the oesophagus. Colorectal locations were examined in 9 studies (33.3%), one of which also included the oesophagus.

### Robotic endoscopic platforms for ESD and their technological maturity

An overview of the robotic endoscopic platforms evaluated across the included studies is presented in Table 2. The most frequently evaluated system (*n* = 6) was the REXTER/ROSE robotic traction system, developed at the Korea University College of Medicine (South Korea). This system has since evolved into the ROBOPERA-TraCloser platform (EndoRobotics, Seoul, South Korea). The MASTER robot and its redesigned successor, the EndoMaster EASE platform (EndoMaster Pte Ltd, Singapore) were evaluated in five studies. This was followed by the FASTER robot and its commercial platform, the EndoFaster system (Robo Medical Technology Co., Ltd., Shenzhen, China) (*n* = 4). The PETH, FLEX, DREAMS, and RAFE systems were each evaluated in two studies. Several other systems were evaluated in single studies (EASE, STRAS v2, GIFTS, ETRS).

**Table 2:** Robotic endoscopic platforms for ESD: study designs, anatomical applications, and technology readiness levels (TRLs)

Robot	Full system name	No. of studies	Study designs	Anatomical locations	TRL Stage^*^
REXTRE / RROSE / ROBOPERA	REXTER: Revolute Joint-based Auxiliary Transluminal Endoscopic Robot ROSE: Robot for Surgical Endoscope ROBOPERA: TraCloser [Dual Gripper]	6	ex vivo, in vivo, clinical	Stomach, colon/rectum	8
MASTER / EndoMaster EASE	MASTER: Master and Slave Transluminal Endoscopic Robot EndoMaster EASE: Endoluminal Access Surgical Efficacy	5	ex vivo, in vivo, clinical	Stomach, colon/rectum	8
FASTER / EndoFaster	Flexible Auxiliary Single-Arm Transluminal Endoscopic Robot	4	ex vivo, in vivo, clinical	Stomach, oesophagus	8
DREAMS	Dual-arm Robotic Endoscopic Assistant for Minimally Invasive Surgery	2	in vivo	Stomach, oesophagus	6–7
RAFE	Robotic-Assisted Flexible Endoscope	2	ex vivo	Stomach	4–5
PETH	Portable Endoscopic Tool Handler	2	ex vivo, in vivo	Stomach	6–7
FLEX	Flex Robotic System	2	ex vivo, clinical	Colon/Rectum	8
EASE	Endoluminal Assistant for Surgical Endoscopy	1	in vivo	Colon/Rectum	6–7
STRAS v2	Single-access Transluminal Robotic Assistant for Surgeons	1	in vivo	Colon/Rectum	6–7
GIFTS	Gastrointestinal Flexible Traction System	1	in vivo	Oesophagus, Colon/Rectum	6–7
ETRS	Endoscopic Therapeutic Robot System	1	ex vivo	Stomach	4–5

^*^TRL classification was assigned pragmatically based on the most advanced experimental context reported for each platform. As TRL boundaries in surgical robotics are not strictly defined, ranges are used for certain devices to reflect judgement-based classification rather than false precision

The developmental maturity of the platforms included in this review was assessed using the TRL scale, a nine-point system that describes the progression of a technology from initial concept to full operational deployment [18]. Most platforms were classified within TRL 4–7, having been validated in ex vivo models or demonstrated in controlled in vivo studies. A smaller number—notably ROBOPERA, EndoMaster, FLEX, and EndoFaster—reached TRL 8, indicating advancement to clinical evaluation or early clinical use.

### Technical architecture and functional classification of robotic endoscopic systems for ESD

Across the included robotic endoscopic systems, several core technical similarities were observed. First, all platforms adopt a teleoperated leader–follower architecture, whereby operator inputs via master controllers are translated into powered distal actuation of the endoscope and/or instruments. Second, tendon- or cable-driven actuation mechanisms represent the dominant transmission strategy across systems. Third, manual endoscope insertion remains standard practice, with robotic deployment occurring after scope positioning. Fourth, all systems employ dedicated operator control interfaces to enable intuitive distal control, with some incorporating haptic or visual feedback enhancements.

Whilst these shared engineering principles establish a common technological foundation, the systems diverge according to their primary operative objective. Functionally, the platforms may be grouped into two broad categories (Table 3).

A. Traction-enhancing robotic adjunct platforms are designed to provide active traction whilst preserving a largely conventional single-instrument ESD workflow. These systems improve tissue exposure and stability without fundamentally altering the structural mechanics of dissection, typically through single-arm add-on modules compatible with standard endoscopes, such as ROBOPERA, EndoFaster, and PETH.
B. Bimanual robotic manipulation platforms enable dual-instrument control and triangulation, thereby modifying operative mechanics through simultaneous tissue manipulation and cutting. By facilitating coordinated traction and dissection, these systems introduce a different procedural paradigm. Within this category, the degree of robotic actuation varies: some systems provide robotic control of both the endoscope and instruments, including STRAS v2, EASE, GIFTS, RAFE, and ETRS. Others provide robotic instrument control with manual endoscope positioning, such as EndoMaster EASE and DREAMS. FLEX represents a distinct configuration within this group, employing robotic endoscope control with manually actuated instruments.

**Table 3:** Functional classification and robotic control characteristics of robotic endoscopic platforms for ESD

Category 1 Traction-enhancing robotic adjunct platforms			
Platform	Uses conventional scope	Robotic scope control	Robotic instrument control
ROBOPERA	✓	✗	✓
EndoFaster	✓	✗	✓
PETH	✓	✗	✓

Category 2 Bimanual robotic manipulation platforms			
Platform	Uses conventional scope	Robotic scope control	Robotic instrument control
EndoMaster EASE	✗	✗	✓
DREAMS	✓	✗	✓
STRAS V2	✗	✓	✓
EASE	✗	✓	✓
GIFTS	✗	✓	✓
RAFE	✓	✓	✓
ETRS	✓	✓	✓
FLEX	✗	✓	✗

Beyond this functional distinction, further structural and engineering heterogeneity is evident. Degrees of freedom (DoFs) and mechanical complexity vary substantially, ranging from relatively simple bending mechanisms to multi-jointed dual-arm configurations with enhanced articulatory capability. Device profile and access strategy also differ, including variation in outer diameter, working-channel configuration, and the requirement for overtubes or dedicated access platforms, all of which influence anatomical reach and procedural feasibility. Finally, systems differ in their workflow integration and deployment architecture, reflecting variation in how robotic components are stabilised, coordinated, and incorporated into the procedural sequence. Full technical specifications are provided in Supplementary Table S1.

### Comparative evaluation of R-ESD and C-ESD in preclinical studies

Thirteen preclinical studies performed head-to-head comparisons of R-ESD and C-ESD. Outcome reporting was inconsistent at the study level, with several studies reporting only a subset of the predefined outcomes. Quantitative outcomes were presented in heterogeneous formats (medians with ranges or interquartile ranges; means with standard deviations), and substantial heterogeneity existed in study design, experimental models, lesion characteristics, and operator experience. These sources of heterogeneity precluded pooled analysis. Outcomes were therefore synthesised descriptively, with R-ESD and C-ESD ranges reported separately, and the direction of effect noted where paired comparisons were available (Table 4), with full study-level data presented in Supplementary Table S2.

**Table 4:** R-ESD versus C-ESD: comparative procedural outcomes in preclinical studies

Outcome	Studies reporting (*n*)	R-ESD range	C-ESD range	Summary of directional effect
Resected specimen area (mm^2^)	8	518–1,570	525–1407	No consistent difference
Submucosal dissection time (min)	12	6–29	4.5–79	11/12 studies favoured R-ESD: 1 favoured C-ESD
Submucosal dissection speed (mm^2^/min)	8	37–240	18–170	All studies favoured R-ESD
En bloc resection (%)	13	100% in 12 studies; 97.5% in 1 study	100% in 9 studies; 50–92.5% in 4 studies	High in both techniques; reductions mainly occurred in novice-operated C-ESD and isolated expert-led cases
Perforation (%)	13	0% in 10 studies; 5–30% in 3 studies	0% in 5 studies; 4.2–60% in 8 studies	Perforation occurred mainly during novice-operated C-ESD; expert-led studies showed only low, isolated events

*R-ESD* robotic-assisted ESD, *C-ESD* conventional ESD

### Procedural efficiency outcomes

Eight studies reported resected specimen area, with overlap between techniques (R-ESD ~ 518–1570 mm^2^; C-ESD ~ 525–1,407 mm^2^), with no consistent directional difference. Twelve studies reported submucosal dissection time (R-ESD 6–29 min; C-ESD 4.5–79 min); eleven studies favoured R-ESD in paired comparisons, whilst one study (Iwasa et al. 2018; RAFE) favoured C-ESD. Eight studies reported submucosal dissection speed (R-ESD 37–240 mm^2^/min vs C-ESD between 18 and 170 mm^2^/min); all paired comparisons favoured R-ESD, with variable magnitude of difference across platforms and operators.

### Procedural success and safety outcomes

Performance outcomes, including *en bloc* resection and perforation, were consistently reported across all thirteen studies. Nine studies achieved 100% *en bloc* resection for both R-ESD and C-ESD. Lower *en bloc* rates appeared mainly with novice-operated C-ESD (Yang et al. 2023:97.5% vs 92.5%; de Moura et al. 2019:100% vs 50%). Additional isolated reductions in C-ESD were reported in Mascagni et al. (2019) (100% vs 90%, in a comparison involving operator experience asymmetry) and Ji et al. (2022) (100% vs 91.7%, same expert operator).

For perforation, most studies reported 0% for both techniques. Higher perforation rates were again concentrated in novice-operated C-ESD (Yang et al. 2023; 0% vs 10%, de Moura et al. 2019; 30% vs 60%, and Kim et al. 2019:10% vs 60%). In Mascagni et al. (2019), R-ESD was performed by a surgeon novice to ESD and C-ESD by an expert endoscopist, resulting in 5% vs 33% perforation. Expert-led studies reported only isolated events, with R-ESD consistently at 0% and C-ESD showing low perforation rates (Ji et al. 2022; 0% vs 4.2%, Kim et al. 2024: 0% vs 12.5%, Yang et al. 2024; 0% vs 20%, and Kim et al. 2024: 0% vs 11%).

### Overview of early clinical evidence on R-ESD

Six clinical studies evaluating R-ESD were identified, spanning IDEAL Stages 1–3. The IDEAL framework classifies evaluation of surgical technologies into staged phases—from early proof-of-concept (Stage 1 and 2a) through exploratory development (Stage 2b) to structured assessment (Stage 3) and long-term surveillance (Stage 4) [19].

Phee et al. (2012) conducted the first human application of a robotic endoscopic system using the MASTER robot in a five-patient case series (IDEAL Stage 2a) with early gastric neoplasia, achieving curative resections without intraoperative adverse events [41]. The second-generation EndoMaster EASE system was later evaluated by Chiu et al. (2025) in a prospective Phase II clinical trial (IDEAL Stage 2b) involving 43 patients with early colorectal neoplasia, mainly in the sigmoid colon and rectum. Technical success was achieved in 37 procedures (86%), with six conversions to C-ESD. Two patients (4.6%) experienced adverse events—one perforation closed with clips, and one delayed bleeding requiring endoscopic haemostasis [43].

The FLEX Robotic Platform was evaluated by Morino et al. (2022) in a prospective case series of 26 patients (IDEAL Stage 2a) with early rectal neoplasia. Fourteen patients underwent robotic-assisted EFTR, and twelve underwent R-ESD. *En bloc* resection was achieved in all cases, with an R0 rate of 84.6%. No intraoperative adverse events occurred; two patients experienced severe post-ESD bleeding, one requiring additional transanal surgery. Conversion to a standard transanal endoscopic operation (TEO) platform was required in six cases to complete dissection or defect closure [42].

Kim et al. (2025) reported initial use of the ROBOPERA system in a two-case series (IDEAL Stage 1), involving a sigmoid laterally spreading tumour and a mid-rectal lesion suspicious for a neuroendocrine tumour. Both procedures achieved *en bloc* and R0 resection without complications [44]. Jeon et al. (2025) subsequently evaluated the system in a 15-patient case series (IDEAL Stage 2a) with early gastric neoplasia. All resections were *en bloc* and R0 with no perforations, though intraoperative bleeding occurred in two patients (13.3%) and delayed bleeding in one patient (6.7%) [46].

Finally, Fan et al. (2025) conducted a multicentre RCT (IDEAL Stage 3) comparing EndoFaster-assisted ESD with C-ESD in 192 patients with early gastric and oesophageal lesions. In the full analysis set, EndoFaster achieved faster dissection speeds in both unadjusted analysis (*p* = 0.00435) and multivariable regression adjusting for lesion size/location, and operator experience (β = − 0.16 cm^2^/min; 95% CI − 0.28 to − 0.04; *p* = 0.008). Safety outcomes were comparable, with no significant differences in muscle-layer injury (OR = 0.95; *p* = 0.88) or operator workload (β = − 1.5; *p* = 0.19). *En bloc* resection rates were non-inferior to C-ESD (98.84% vs. 98.13%), and no device-related adverse events occurred [45].

## Discussion

ESD offers a curative, organ-preserving approach to early-stage GI cancers, yet its technical complexity results in a steep learning curve and elevated procedural risk, restricting widespread adoption beyond high-volume specialist centres [47–50]. These challenges have motivated growing interest in robotic endoscopic systems, building on the broader success of robotic technologies in overcoming technical constraints in selected surgical operations [51, 52]. Within this context, ESD has emerged as a primary target application for endoscopic robotics [53], and a growing body of preclinical and early clinical evidence has begun to characterise the potential role of robotic assistance in this setting. The findings of this review should nonetheless be interpreted as exploratory signals of potential benefit rather than as evidence of established clinical superiority over conventional techniques.

Despite expanding interest, R-ESD remains an emerging field, with substantive progress occurring only in recent years. The predominance of feasibility-oriented studies reflects an evidence base still focussed on establishing technical viability rather than sustained clinical implementation. When considered through the lens of technological maturity, most platforms remain at intermediate developmental stages—validated primarily in ex vivo or in vivo settings—with only a small number having progressed to clinical evaluation. This pattern underscores the translational gap between proof-of-concept demonstration and routine clinical adoption.

The geographical concentration of studies in East Asia reflects longstanding differences in ESD adoption, disease epidemiology, and technical expertise compared with Western centres [54–57]. This has likely shaped both the pace and direction of technological development in this field. The predominance of gastric applications is consistent with higher gastric cancer prevalence in East Asia, which drives greater clinical and technological focus on gastric ESD [58]. Gastric models also offer practical advantages as a test environment—including a larger, more compliant working space and lower perforation risk than colonic preparations—supporting their established role in ESD training [59, 60], and their preferential use for early-stage robotic device evaluation.

Comparative preclinical evidence suggests that R-ESD is associated with improved procedural efficiency compared with conventional techniques. The near-uniform direction of effect across paired comparisons—observed in both traction-enhancing adjunct and bimanual robotic manipulation platforms—suggests a consistent directional trend rather than an effect confined to a single platform or system class. This consistency likely reflects the shared functional benefits of robotic assistance during the most technically demanding phase of ESD: improved traction, more stable tissue exposure, and enhanced instrument control. However, these comparisons were not standardised across platforms, operators, or lesion complexity, and robust comparison of effect magnitude at the platform or class level remains constrained by this heterogeneity and by variability in outcome selection, definition, and reporting. Greater standardisation of experimental models and core outcome measures would strengthen evaluation and cross-platform comparability of emerging robotic ESD technologies.

Importantly, these efficiency gains do not appear to compromise resection quality: resected specimen areas were comparable, and *en bloc* resection rates were high across studies, with reductions primarily observed in novice-operated C-ESD. Perforation rates were likewise low overall, with higher rates concentrated in novice-operated C-ESD, suggesting that robotic assistance may help mitigate operator-related risks. In expert-led studies, perforation events were infrequent for both approaches, suggesting that the incremental safety benefit of robotics diminishes as expertise increases. Taken together, these findings suggest that operator experience may function as an important effect modifier, with robotic assistance conferring its greatest relative benefit during the learning phase rather than in already proficient hands. However, not all studies were designed specifically to isolate operator experience as a causal variable, and these findings should therefore be interpreted with caution. This has important implications for how robotic ESD systems are positioned—as potential enablers of safer skill acquisition and broader dissemination of ESD beyond high-volume specialist centres.

Clinical evidence for R-ESD remains early and heterogeneous. EndoFaster is currently the most advanced platform, having reached IDEAL Stage 3 evaluation through a multicentre RCT—though it should be noted that IDEAL Stage 3 represents structured comparative assessment rather than definitive evidence of routine clinical benefit. In contrast, other systems remain within IDEAL Stages 1–2b and therefore cannot yet be considered ready for broad adoption. Across platforms, early human use appears feasible, with consistently high *en bloc* and R0 resection rates and low rates of adverse events, suggesting no new safety concerns attributable to robotic assistance. The only RCT to date demonstrated that robotic assistance improves procedural performance—particularly dissection speed—whilst achieving safety and oncological outcomes comparable to C-ESD [45]. However, this study primarily evaluated technical performance endpoints rather than patient-centred or long-term oncological outcomes. The EndoFaster also functions as a traction-enhancing adjunct, and hence, its findings may not be directly extrapolated to bimanual robotic manipulation platforms. Whether robotic assistance will ultimately confer benefits beyond procedural efficiency—particularly in terms of oncological outcomes or patient-centred endpoints—remains an important unanswered question.

Across the robotic endoscopic systems included in this review, substantial heterogeneity persists in platform design and implementation, reflecting a field that has not yet converged on a dominant technical solution. The platforms can nonetheless be broadly classified into traction-enhancing adjunct systems and bimanual robotic manipulation systems, a distinction that carries important implications for procedural workflow and system integration. Traction-enhancing adjunct platforms integrate into an otherwise conventional ESD workflow, adding active tissue exposure without fundamentally altering how the procedure is performed—their relative simplicity suggesting a more accessible learning curve and lower barrier to adoption. Bimanual robotic manipulation platforms, by contrast, introduce a different operative paradigm—enabling simultaneous traction and dissection through dual-instrument control—which may offer greater procedural capability but demands more substantial retraining and workflow reorganisation. Beyond this functional distinction, several shared constraints are evident across systems. Device bulk and limited miniaturisation restrict manoeuvrability and anatomical reach. Robotic assistance moreover remains commonly restricted to submucosal dissection, with several systems still requiring manual endoscope or instrument manipulation. Prototype-level reliability, sterility and setup burdens, and restricted instrument portfolios further limit clinical readiness across most platforms.

Beyond technical performance, future adoption of robotic endoscopic systems will also depend on economic, workflow, and regulatory considerations. These platforms introduce additional capital costs, maintenance requirements, and training demands that must be justified by demonstrable clinical or efficiency benefits. Integration into existing endoscopy units may require changes to room setup, staffing models, and procedural logistics. Furthermore, regulatory approval pathways for complex robotic systems can be lengthy and resource-intensive, particularly as platforms evolve iteratively across development stages. Addressing these implementation barriers will be as important as continued technical refinement in determining whether robotic ESD transitions from a specialist research tool to a routinely adopted clinical technology.

This review has several limitations. First, eligibility criteria were restricted to robotic endoscopic systems evaluated specifically for ESD and reporting established ESD-related outcomes. Consequently, emerging robotic endoscopic technologies may have been excluded if they had not yet undergone formal ESD evaluation or were reported under alternative procedures or engineering-focussed endpoint, and the findings may therefore not fully reflect the breadth of ongoing innovation in endoscopic robotics. Second, data charting was conducted by a single reviewer, which—whilst acceptable for a scoping review—may have increased the risk of extraction error and limited independent verification of reported outcomes, including the narrative interpretation of outcome ranges and direction of effect. Third, substantial methodological heterogeneity across studies precluded pooled analysis, and observed advantages of robotic endoscopic systems should therefore be interpreted as signals of potential benefit rather than definitive evidence of superiority. Finally, assignment of TRL and IDEAL stages relied on interpretation of published descriptions and involved subjective judgement.

R-ESD remains an evolving field, with most platforms still at intermediate stages of validation and only a small number having reached early clinical evaluation. Preclinical comparative evidence signals improved procedural efficiency without compromising resection quality or safety, with benefits most apparent in less experienced operators. Clinical evidence remains limited to early-phase studies demonstrating feasibility and short-term safety and does not yet establish superiority over C-ESD. No dominant technical solution has emerged, and persistent design heterogeneity, workflow limitations, and implementation barriers indicate that the field has not yet reached maturity. Future progress will depend on improved workflow integration, standardised outcome reporting, evaluation of training pathways, cost-effectiveness analyses and robust comparative clinical trials—collectively determining whether robotic ESD transitions from a promising investigational technology to a routinely adopted clinical tool.

## Supplementary Information

Below is the link to the electronic supplementary material.

Supplementary file1 (PDF 36 KB)

Supplementary file2 (DOCX 41 KB)

Supplementary file3 (DOCX 40 KB)
