# Supplementary material for: Robotic-assisted endoscopic submucosal dissection: a scoping review of preclinical and early clinical evidence
Source: Surg Endosc. 2026 Jul 1;40(8):6409–21. doi: 10.1007/s00464-026-12980-6 (PMC13457265; doi:10.1007/s00464-026-12980-6)
Supplement: Supplementary file 1 [file 464_2026_12980_MOESM1_ESM.pdf]

Midline

Ovid MEDLINE(R) ALL <1946 to February 19, 2025>

1 exp Robotics/ 47389

2 (Robot* or Telerobot*).mp. [mp=title, book title, abstract, original title, name of substance word, subject heading word, floating sub-heading word, keyword heading word, organism supplementary concept word, protocol supplementary concept word, rare disease supplementary concept word, unique identifier, synonyms, population supplementary concept word, anatomy supplementary concept word] 93556

3 1 or 2 93556

4 exp Endoscopic Mucosal Resection/ 4615

5 (endoscopic submucosal dissection* or ESD).mp. [mp=title, book title, abstract, original title, name of substance word, subject heading word, floating sub-heading word, keyword heading word, organism supplementary concept word, protocol supplementary concept word, rare disease supplementary concept word, unique identifier, synonyms, population supplementary concept word, anatomy supplementary concept word] 10141

6 4 or 5 11676

7 3 and 6 112

Embase

Embase Classic+Embase <1947 to 2025 February 19>

1 exp Robotics/ 50309

2 (Robot* or Telerobot*).mp. [mp=title, abstract, heading word, drug trade name, original title, device manufacturer, drug manufacturer, device trade name, keyword heading word, floating subheading word, candidate term word] 143038

3 1 or 2 143128

4 exp Endoscopic Mucosal Resection/ 10176

5 (endoscopic submucosal dissection* or ESD).mp. [mp=title, abstract, heading word, drug trade name, original title, device manufacturer, drug manufacturer, device trade name, keyword heading word, floating subheading word, candidate term word] 21329

6 4 or 5 27760

7 3 and 6 376

Cochrane Library

Search Name: Robotic ESD Date Run: 20/02/2025 12:38:38 Comment:

ID Search Hits

#1 MeSH descriptor: [Robotics] explode all trees 2042

#2 Robot* OR Telerobot* 8590

#3 #1 OR #2 8590

#4 MeSH descriptor: [Endoscopic Mucosal Resection] explode all trees 230

#5 endoscopic submucosal dissection* OR ESD 1550

#6 #4 OR #5 1629

#7 #3 AND #6 34

Web of Science

(ALL=(Robot* OR telerobot*)) AND ALL=(endoscopic submucosal dissection* OR esd)

IEEE Xplore

("All Metadata":robot* OR telerobot*) AND ("All Metadata":endoscopic submucosal dissection* OR esd)

Scopus

(TITLE-ABS-KEY (endoscopic AND submucosal AND dissection* OR esd) AND TITLE-ABS-KEY (robot* OR telerobot*))
