# Supplementary material for: Robotic-assisted endoscopic submucosal dissection: a scoping review of preclinical and early clinical evidence
Source: Surg Endosc. 2026 Jul 1;40(8):6409–21. doi: 10.1007/s00464-026-12980-6 (PMC13457265; doi:10.1007/s00464-026-12980-6)
Supplement: Supplementary file 2 [file 464_2026_12980_MOESM2_ESM.docx]

| **Supplementary Table S1. Robotic Endoscopic Systems for ESD: Technical Specifications** | | | | | | | | | | |
| --- | --- | --- | --- | --- | --- | --- | --- | --- | --- | --- |
| **Robot Name** | **Developer** | **System Architecture** | **Degrees of Freedom (DoFs)** | **Actuation Mechanism** | **Control Mode** | **Operators** | **User Interface** | **Endoscope Compatibility** | **Overtube or Access Device** | **Deployment** |
| **ROBOPERA** | EndoRobotics Co., Ltd. (Korea) | Tip-mounted, single-arm, robotic add on system | 5 DoFs (bending x2, wrist articulation, rotation, independent jaws) | Cable-driven articulated alligator arm | Manual endoscope; robotic arm | 2 | Main and auxiliary handheld controllers via driver unit | Yes | Yes; 25-cm oesophageal overtube (Guardus) | Tip-mounted arm introduced via overtube |
| **EndoMaster EASE** | EndoMaster Pte Ltd. (Singapore) | Dual-arm robotic system integrated into a proprietary multi-channel endoscope | 10 DoFs per arm | Tendon–sheath mechanism | Manual endoscope; robotic instruments | 1 | Console with joystick controllers for dual-arm teleoperation and integrated endoscopic imaging | No; proprietary endoscopic platform | No | Endoscope docked to console with robotic arms deployed through dedicated channels |
| **EndoFaster** | Robo Medical Technology Co, Ltd, (China) | Add-on robotic system with reusable control unit and disposable distal actuator | 4 DoFs (distal add-on robotic arm) | Tendon–sheath continuum actuator driven by lead-screw slider mechanism | Manual endoscope; robotic instrument | 1 | 6-DoF master manipulator with trigger control | Yes; hood-mounted attachment | No | Distal actuator mounted on endoscope and inserted manually |
| **DREAMS** | Qilu Hospital of Shandong University (China) | Miniature dual-arm robotic system integrated with a conventional dual-channel endoscope | Knife: 3 DoFs; Forceps: 5 DoFs; Robotic arm: 6 DoFs | Flexible Parallel Continuum Wrist (FPCW) using NiTi wires with motorised endoscope positioning | Manual  endoscope; robotic  instruments | 2 | Dual master controllers for bimanual instrument manipulation with integrated buttons, plus a separate panel for camera and endoscope orientation control | Yes; dual-channel therapeutic endoscopes | No | Manual endoscope insertion followed by deployment of robotic instruments through working channels |
| **RAFE** | Kyushu University (Japan) | Modular robotic system with actuation of a conventional endoscope and dual flexible robotic instruments | Endoscope: 4 DoFs (+ auxiliary actuators); Instruments: 2 DoFs + end-effector actuation | Tendon-driven endoscope actuation and Bowden-cable instrument actuation | Robotic control of endoscope and instruments | 1 | Console with dual joysticks for instrument bending, sliders for insertion, thumb knobs for end-effector actuation, and a separate joystick for endoscope control | Yes; external tube added to provide a second instrument channel | No | Manual endoscope insertion followed by fixation in robotic holder |
| **PETH** | KAIST Institute & The Catholic University of Korea, with contributions from Roen Surgical, Inc. (Korea) | Add-on teleoperated flexible instrument module mounted on a conventional endoscope | Robotic arm: 2 bending DoFs; 1 instrument channel (2.8 mm); combined endoscope + module diameter: 15 mm | Tendon-driven bending arm | Manual endoscope; robotic instrument | 2 | Master control device with thumbstick-based arm control; graphical display/simulator showing real-time arm configuration | Yes | Yes;  custom 23 mm overtube | Add-on arm mounted on endoscope and introduced via overtube |
| **FLEX** | Medrobotics Corporation (USA) | Proprietary robotic endoscopic platform with manual articulating instruments | Not stated | Tendon-driven robotic endoscope with manually actuated flexible instruments | Robotic endoscope; manual instruments | 1 | Console with joystick for endoscope control with pistol-grip manual instruments (3D HD visualisation) | No; proprietary robotic endoscope (Flex® Drive) | Yes; rectal port | Inserted via rectal port; scope advanced to target (reported reach ≤25 cm from anal verge); instruments deployed through working channels |
| **EASE** | Research Institute Against Digestive Cancer (IRCAD) (France) | Modular robotic system with actuation of a dedicated endoscope and dual telemanipulated instruments | Instruments: 3 DoFs each; endoscope with 4-way tip control plus rotation and translation | Tendon-driven actuation for endoscope and instruments | Robotic control of endoscope and instruments | 1 | Master console with dual joysticks and thumb controls for endoscope and instrument manipulation; dual-screen endoscopic view and graphical mapping | No; dedicated 16 mm, 53.5 cm robotic endoscope with three working channels | Yes; GelPOINT transanal access platform | Endoscope introduced via GelPOINT and docked to slave cart |
| **STRAS v2** | IRCAD & ICube Laboratory (France), supported by Karl Storz Endoskope (Germany) | Robotic system based on a modified Anubiscope™/Isis-scope® endoscopic platform | Instruments: ~3–4 DoFs each + grasper (open/close); endoscope with multidirectional tip and axial motion | Tendon-driven actuation for endoscope and instruments | Robotic control of endoscope and instruments | 2 | Master console with dual hand controllers; thumb switches for endoscope deflection; grasper triggers; joystick for endoscopic navigation | No; modified Anubiscope™/Isis-scope® (18 mm, 50 cm; dual 4.2 mm channels + 2.8 mm auxiliary channel) | No | Manual endoscope insertion followed by docking to motorised cart and deployment of robotic instruments |
| **GIFTS** | Tianjin University (China) | Robotic endoscopic system with variable-stiffness insertion tube and two flexible robotic instruments | Endoscope: 4 DoFs; Forceps: 5 DoFs; Knife: 4 DoFs | Tendon-driven actuation with variable stiffness control using Nitinol tension wires | Robotic control of endoscope and instruments | 1 | Dual haptic controllers providing force feedback and visual input for master–slave control of the variable-stiffness system | No; proprietary robotic endoscope (10 mm diameter, dual 3.2 mm channels) | No | Manual endoscope insertion with in situ stiffness adjustment followed by deployment of robotic instruments |
| **ETRS** | University of Occupational and Environmental Health & Kyushu Institute of Technology (Japan) | Robotic system with actuation of a modified conventional endoscope | Not stated | Tendon-driven robotic actuation integrated with a modified endoscope | Robotic control of endoscope and instruments | 1 | Two haptic master devices for tool control, a dedicated endoscope control unit, foot-switch auxiliary controls, and a haptic-enabled catheter/drug injection module | Yes; modified Olympus GIF-XQ240 | Yes; 20 mm overtube | Manual endoscope insertion via overtube |
