# Supplementary material for: Robotic-assisted endoscopic submucosal dissection: a scoping review of preclinical and early clinical evidence
Source: Surg Endosc. 2026 Jul 1;40(8):6409–21. doi: 10.1007/s00464-026-12980-6 (PMC13457265; doi:10.1007/s00464-026-12980-6)
Supplement: Supplementary file 3 [file 464_2026_12980_MOESM3_ESM.docx]

| **Supplementary Table S2. R-ESD versus C-ESD in Preclinical Models: Study Characteristics and Comparative Outcomes** | | | | | | | | | | | | | | | | | |
| --- | --- | --- | --- | --- | --- | --- | --- | --- | --- | --- | --- | --- | --- | --- | --- | --- | --- |
| **Study** | **Robotic Platform** | **Experimental Design** | | | | | | **Outcomes^*^** | | | | | | | | | |
|  |  | **Design** | **Target Organ** | **No. of Pigs/Specimens (Lesion Location)** | **No. of Operators (Experience)** | **No. of ESDs** | | **Resected Specimen Area (mm^2^)** | | **Submucosal Dissection Time (min)** | | **Submucosal Dissection Speed (mm^2^/min)** | | **En Bloc Resection (%)** | | **Perforation (%)** | |
|  |  |  |  |  |  | **R** | **C** | **R** | **C** | **R** | **C** | **R** | **C** | **R** | **C** | **R** | **C** |
| **Yang et al. (2022)** | **FASTER** | *ex vivo* | Stomach | NA (Anterior and posterior walls of the antrum (8 vs 8; 10 vs 10)) | 1 (ESD expert; ~200 ESDs, 10 prior FASTER cases) | 18 | 18 | 699 (546-2270) | 1010 (723-2196) | 7 (4-13) | 13 (8-20) | 148.6 ± 53 | 97.0 ± 37 | 100 | 100 | 0 | 0 |
| **Hwang et al. (2020)** | **PETH** | *ex vivo* | Stomach | 17 stomachs (6 vs 3 per site (Body GC, Body LC, Antrum GC, Antrum LC, Angle)) | 1 (ESD expert; >800 ESDs, no prior robotic experience) | 30 | 15 | 1119 (908-1484) | 825 (589-1018) | 12 (7-15) | 21 (14-30) | 122.3 ± 76.5 | 47.5 ± 26.9 | 100 | 100 | 0 | 0 |
| **Iwasa et al. (2018)** | **RAFE** | *ex vivo* | Stomach | NA | 1 (ESD expert; no prior robotic experience) | 6 | 6 | NA | | 9.5 (6–11) | 4.5 (3–6) | NA | NA | 100 | 100 | 0 | 0 |
| **Mascagni et al. (2019)** | **EASE** | *in vivo* | Colon | 10 pigs (15–35 cm from anal verge) | 2 (R-ESD by surgeon novice to ESD; C-ESD by expert endoscopist) | 20 | 10 | 1573 ± 808 | 1407 ± 409 | 29.42 ± 14.29 | 43.43 ± 12.55 | 57.05 ± 29.42 | 35.21 ± 16.20 | 100 | 90 | 5 | 33 |
| **Yang et al. (2023)** | **FASTER** | *ex vivo* | Stomach | 80 stomachs (Two adjacent lesions on posterior antral wall) | 4 (ESD novices; two per group, <5 *ex vivo* ESDs, no *in vivo* ESDs) | 40 | 40 | NA | | 13.9 ± 5.5 | 23.1 ± 11 | 240 (170-360) | 170 (120 - 220) | 97.5 | 92.5 | 0 | 10 |
| **de Moura et al. (2019)** | **FLEX** | *ex vivo* | Colon | NA | 5 (ESD novices; <5 *ex vivo* ESDs, no *in vivo* ESDs; Group 1: 3 performed 2 C-ESDs then 2 R-ESDs; Group 2: 2 performed 2 R-ESDs then 2 C-ESDs) | 10 | 10 | NA | | 27.8 ± 18.38 | 79.4 ± 30.70 | NA | | 100 | 50 | 30 | 60 |
| **Ho et al. (2010)^†^** | **MASTER** | *in vivo* | Stomach | 5 pigs (Location not specified) | 1 (ESD expert; >15 years interventional endoscopy, experienced with MASTER, involved in its development since 2004) | 5 | 5 | NA | | 16.2 (3-29) | 18.6 (9-34) | NA | | 100 | 100 | 0 | 0 |
| **Kim et al. (2019)^‡^** | **REXTER** | ex vivo | Stomach | 20 (NA) | 3 (ESD experts, >500 ESDs) | 10 | 10 | NA | | NA | | NA | | 100 | 100 | 0 | 0 |
| **Kim et al. (2019)^‡^** | **REXTER** | ex vivo | Stomach | 20 (NA) | 3 (ESD novices, <10 ESDs) | 10 | 10 | NA | | NA | | NA | | 100 | 100 | 10 | 60 |
| **Ji et al. (2022)** | **FASTER** | *in vivo* | Oesophagus, Stomach | 6 pigs (Anterior/posterior oesophagus (mid, lower) and gastric body/antrum (GC, LC)) | 1 (ESD expert; ~300 ESDs, 20 FASTER-assisted *ex vivo* cases) | 24 | 24 | 830 | 810 | 11.4 (9.0-13.3) | 25.7 (17.5-26.5) | NA | | 100 | 91.7 | 0 | 4.2 |
| **Kim et al. (2024)** | **PETH** | *in vivo* | Stomach | 4 pigs (8 gastric sites (upper/lower anterior, posterior, LC, GC); 1 C-ESD and 2 R-ESDs per site) | 1 (ESD expert; 10 years, >1000 ESDs) | 16 | 8 | 1280.4 (1180.2–1704.1) | 1363.0 (1268.1–1656.4) | 8.33 ± 4.03 | 14.09 ± 8.6 | 234 ± 108 | 132 ± 54 | 100 | 100 | 0 | 12.5 |
| **Yang et al. (2024)** | **DREAMS** | *in vivo* | Oesophagus, Stomach | 8 pigs (12 gastric (GC) and 8 oesophageal (left wall) lesions per group) | 1 (ESD expert; ~200 ESDs, 10 DREAMS-assisted *ex vivo* cases) | 20 | 20 | 816 ± 190 | 789 ± 340 | 9.80 ± 4.2 | 12.44 ± 6.8 | 94 ± 30 | 82 ± 50 | 100 | 100 | 0 | 20 |
| **Kim et al. (****2024)^†^** | **Non-specified^§^** | *ex vivo* | Colon, Rectum | 6 colons (Procedures equally distributed across ascending, transverse, and descending zones in both R-ESD and C-ESD groups) | 1 (ESD expert with; >300 ESDs) | 18 | 18 | 518.4±27.2 | 525.5±23.8 | 14.9±4.1 | 30.9±8.3 | 36.8±9.2 | 18.1±4.7 | 100 | 100 | 0 | 11 |
| **Kim et al. (2025)** | **ROBOPERA** | *in vivo* | Stomach | 6 pigs (2 gastric sites (GC of antrum and GC of mid-body); 1 C-ESD and 1 R-ESD per site) | 1 (ESD expert; >500 ESDs) | 12 | 12 | 928.1 ± 74.5 | 937.2 ± 54.8 | 6.1 ± 2.1 | 11.8 ± 1.3 | 157.6 ± 33.3 | 91.4 ± 23.7 | 100 | 100 | 0 | 0 |
| *Abbreviations:* R-ESD or R = robotic-assisted ESD; C-ESD or C = conventional ESD; NA = Not provided or not directly reported.  ^*^ Outcomes were reported as mean ± SD, median (IQR), or percentages. Resected specimen area was generally reported in mm², dissection time in minutes, and dissection speed in mm² per minute. To ensure consistency in data presentation, unit conversions were performed only when necessary. Specifically, specimen area was converted from cm² to mm², dissection speed from cm²/min to mm²/min or from mm²/s to mm²/min, and dissection time from seconds to minutes.  ^†^ Comparative only in one component of a mixed *ex vivo/in vivo* study.  ^‡^ Kim et al. (2019) is shown in two rows because the study reported outcomes separately for expert and novice operators.  ^§^ Device name not specified in the publication; however, it is part of the REXTER/ROSE series developed by the same research group. | | | | | | | | | | | | | | | | | |
